# dNK3 cells in normal pregnancy and recurrent pregnancy loss: from molecular identity to functional imbalance

**DOI:** 10.3389/fimmu.2026.1822205

**Published:** 2026-05-13

**Authors:** Lidan Liu, Zhao Zhang, Qianyi Huang, Bo Liu, Hongbo Wu

**Affiliations:** 1 The First Affiliated Hospital of Guangxi Medical University, Nanning, China; 2 Qinzhou Maternity and Child Healthcare Hospital, Qinzhou, China

**Keywords:** decidual natural killer cells, dNK3, maternal-fetal interface, recurrent pregnancy loss, single-cell transcriptomics

## Abstract

**Background:**

Decidual natural killer (dNK) cells constitute approximately 70% of first-trimester decidual leukocytes and play critical roles in immune tolerance, angiogenesis, and trophoblast invasion. Single-cell RNA sequencing has revealed substantial heterogeneity within the dNK population, identifying three major subsets—dNK1, dNK2, and dNK3—with distinct transcriptomic profiles and predicted functions. dNK3 Characteristics: dNK3 cells are characterized by a CD160^+^KLRB1^+^CD103^+^CD39^−^ surface phenotype, T-bet-high/Eomes-intermediate transcription factor profile, and phenotypic resemblance to intraepithelial type 1 innate lymphoid cells (ieILC1). These cells demonstrate the highest effector capacity among dNK subsets, producing multiple cytokines (CCL5, XCL1, IFN-γ, GM-CSF) following stimulation. Predicted ligand-receptor interactions include CCL5–CCR1 with extravillous trophoblasts, XCL1–XCR1 with dendritic cells, and inhibitory axes through KLRB1–CLEC2D and TIGIT–PVR. Notably, dNK3 abundance undergoes dynamic changes across gestation and shows distinct spatial distribution within decidual compartments.

**Clinical relevance:**

Multiple independent studies have identified a reproducible dNK1-down/dNK3-up shift in recurrent pregnancy loss (RPL), with dNK3 cells showing IFNG upregulation at chromatin, transcriptional, and protein levels. This subset imbalance positions the dNK1/dNK3 ratio as a candidate diagnostic biomarker and identifies potential therapeutic targets including M-CSF supplementation, TGF-β pathway modulation, and iPSC-derived dNK cell therapy.

**Conclusions:**

While dNK3 represents a promising focus for reproductive immunology and RPL, the current evidence base remains insufficient for clinical translation. Critical questions regarding causality, cross-study comparability, and the dNK3/ieILC1 developmental relationship require resolution through prospective cohorts and rigorous functional validation.

## 1 Introduction

### 1.1 The decidual immune microenvironment

The establishment and maintenance of human pregnancy require remarkable immunological adaptation at the maternal-fetal interface (1, 2). The decidua — the specialized endometrial tissue that forms during pregnancy — harbors a unique immune cell repertoire dominated by natural killer (NK) cells, which constitute approximately 70% of decidual leukocytes during the first trimester (3, 4). Unlike their cytotoxic counterparts in peripheral blood, decidual NK (dNK) cells predominantly exhibit a CD56^bright^ CD16^–^ phenotype (5–7). These cells are primarily involved in immune tolerance, angiogenesis, and trophoblast invasion support rather than target cell killing (5, 7). Early immunohistochemical studies by Koopman et al. established that dNK cells represent a distinct NK subset with immunomodulatory functions, markedly different from peripheral blood NK cells (4).Studies in murine models have further demonstrated the critical role of dNK cells: NK cell deficiency leads to impaired spiral artery remodeling, inadequate placentation, and adverse pregnancy outcomes (3, 8). The KIR (killer cell immunoglobulin-like receptor) repertoire of uterine NK cells undergoes pregnancy-specific changes, further underscoring the specialized nature of these cells (9).In addition, natural killer T (NKT) cells at the maternal-fetal interface contribute to pregnancy maintenance through Th2 cytokine production and immune regulation (6).

### 1.2 Origin of decidual NK cells

The ontogeny of dNK cells is now recognized as a convergent, context-dependent process encompassing at least three non-mutually exclusive developmental trajectories rather than a single linear pathway. First, peripheral CD56^bright^CD16^−^ NK cells—particularly CXCR4-expressing subsets—are selectively recruited to the decidua along chemotactic gradients established by trophoblast- and stromal-derived CXCL12 (10–12). Second, tissue-resident CD34^+^ hematopoietic precursors within the uterine mucosa can undergo local commitment to the dNK lineage under the combinatorial influence of an IL-15-enriched decidual cytokine milieu and stromal-derived instructive signals (11, 13). Third, circulating cytotoxic CD56^dim^CD16^+^ NK cells may be phenotypically and functionally reprogrammed upon entering the uterine microenvironment; TGF-β, in particular, has been demonstrated to orchestrate the conversion of these cells toward a CD16^−^, hypocytotoxic, decidual-like state endowed with enhanced tissue-remodeling capacity (13). Unbiased single-cell transcriptomic profiling has further substantiated the view that the dNK compartment comprises a heterogeneous assembly shaped by the concurrent operation of local differentiation and peripheral recruitment, rather than by any single precursor source (11, 14). Notably, dNK cells do not appear to be regulated primarily through direct classical steroid receptor signaling. Instead, progesterone and other endocrine inputs are thought to be transduced indirectly through decidual stromal cells, which serve as critical intermediaries by translating hormonal cues into local IL-15-, TGF-β-, and chemokine-dependent programs that collectively govern dNK cell homing, maturation, and functional specialization (11–13).

### 1.3 Functional repertoire of decidual NK cells

**Figure 1:**
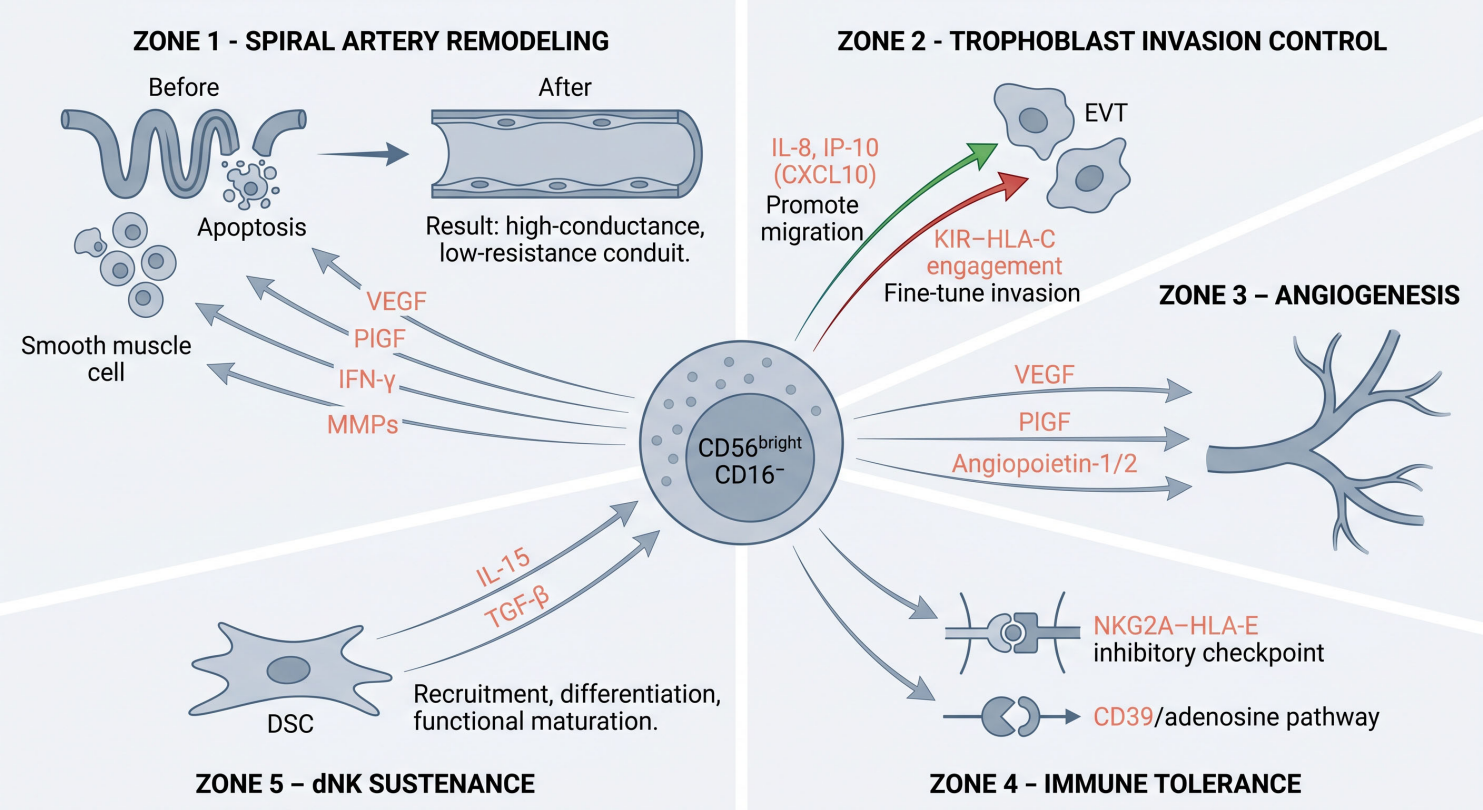
Functional repertoire of decidual NK cells in early pregnancy. dNK cells mediate five key functions: spiral artery remodeling (VEGF, PlGF, IFN-γ, MMPs), trophoblast invasion control (IL-8/CXCL10 promotion vs KIR–HLA-C fine-tuning), angiogenesis (VEGF, PlGF, Ang-1/2), immune tolerance (NKG2A–HLA-E, CD39/adenosine), and stromal cell-dependent maintenance (IL-15, TGF-β).

Functionally, dNK cells execute a suite of specialized effector programs that are indispensable for the establishment and maintenance of a successful pregnancy (**Figure 1**). First, dNK cells initiate the remodeling of uterine spiral arteries—even prior to the arrival of extravillous trophoblast (EVT) cells—by secreting vascular endothelial growth factor (VEGF), placental growth factor (PlGF) (10), interferon-γ (IFN-γ), and matrix metalloproteinases (MMPs) (12, 15), thereby promoting vascular smooth muscle cell apoptosis and converting these vessels into high-conductance, low-resistance conduits capable of sustaining adequate uteroplacental perfusion. Second, dNK cells exert bidirectional control over trophoblast invasion: on the one hand, they facilitate EVT migration through the secretion of the chemokines IL-8 and IP-10 (CXCL10); on the other, they fine-tune the depth and extent of invasion through the engagement of killer immunoglobulin-like receptors (KIRs) with HLA-C on EVT (10, 14, 16). Third, dNK cells constitute the predominant cellular source of pro-angiogenic mediators within the decidua, including VEGF, PlGF, and angiopoietin-1/2, thereby driving the vascularization of the nascent placenta (10, 12). Fourth, dNK cells contribute to the establishment of immune tolerance at the maternal–fetal interface through the NKG2A–HLA-E inhibitory checkpoint and the CD39/adenosine immunosuppressive pathway (11, 12, 14). Finally, dNK cells are sustained by decidual stromal cells through IL-15- and TGF-β-dependent signaling, which collectively supports their recruitment, differentiation, and functional maturation within the decidual microenvironment (12, 13).

### 1.4 Single-cell transcriptomics and dNK heterogeneity

Single-cell RNA sequencing (scRNA-seq) has substantially advanced the understanding of dNK cell biology by revealing substantial heterogeneity within this population. Vento-Tormo et al. (14) profiled over 70,000 cells from the early maternal-fetal interface and identified three major dNK subsets—dNK1, dNK2, and dNK3—each with distinct transcriptomic profiles, surface markers, and predicted functions (14). This classification has been validated through mass cytometry (CyTOF) (17), additional scRNA-seq studies (18, 19), and functional experiments (20). CellPhoneDB analysis revealed that each dNK subset engages different cellular partners through specific communication pathways (14, 21).It should be noted, however, that different research groups have used varying clustering approaches and resolution parameters: while Vento-Tormo et al. identified three major subsets, other studies have reported four or five clusters depending on analytical methods. Direct comparisons between clusters from different studies should therefore be interpreted cautiously, and subset identification should be based on marker expression rather than cluster number alone.

### 1.5 dNK3: an emerging subset of clinical interest

#### 1.5.1 Molecular and functional characteristics

Of the three canonical dNK subsets, dNK3 cells are of particular interest given their distinctive molecular profile and observed association with pregnancy pathology.

However, it is important to distinguish between correlation and causation. While altered dNK3 proportions are consistently observed in pregnancy complications, whether these changes are causative, consequential, or coincidental remains to be determined. These cells are defined by expression of CD160, KLRB1 (CD161), and CD103, with notably absent expression of CD39 and KIR receptors (14, 17). Functionally, dNK3 cells demonstrate the strongest effector responses among dNK subsets, producing multiple cytokines (CCL5, XCL1, IFN-γ, GM-CSF) following *in vitro* stimulation (17). The phenotypic similarity between dNK3 and intraepithelial ILC1 (ieILC1) found in other mucosal tissues raises questions about their developmental relationship and position within the broader innate lymphoid cell (ILC) family (17, 22–24).

#### 1.5.2 Clinical associations with recurrent pregnancy loss

Several independent scRNA-seq analyses of decidual tissue from recurrent pregnancy loss (RPL) patients have converged on a consistent observation: dNK1 proportions decrease while dNK3 proportions increase (18, 19).Although this pattern appears reproducible, the causal relationship between altered dNK subset distribution and pregnancy loss remains to be determined.

### 1.6 Scope and objectives of this review

This review synthesizes current knowledge of dNK3 cell biology, covering their molecular identity, physiological functions, gestational dynamics, and pathological associations. This review addresses four key questions: (i) How are dNK3 cells defined at the molecular, transcriptional, and functional levels? (ii) How do dNK3 cells contribute to normal pregnancy through ligand-receptor interactions, signaling pathways, and effector functions? (iii) What evidence exists for dNK3 alterations in RPL, and what mechanisms might underlie the observed dNK1-down/dNK3-up shift? (iv) What diagnostic and therapeutic opportunities does dNK3 research present?

## 2 Molecular identity of dNK3 cells

### 2.1 Surface marker profile: a multi-level classification system

dNK3 cells can be distinguished from dNK1 and dNK2 by their surface marker expression. These cells express multiple activation markers including CD160, KLRB1 (CD161), ITGAE (CD103), ITGB2 (CD18), TIGIT, NKG2D, and CD69. In contrast, they lack CD39 (ENTPD1) and show little or no expression of inhibitory receptors such as KIR family members (KIR2DL1, KIR2DL3, KIR3DL1) or LILRB1 (14, 17). This places dNK3 at one end of the functional spectrum, opposite to dNK1, which expresses CD39, multiple KIRs, LILRB1, and high levels of NKG2A (4, 9, 14). Notably, KIR–HLA-C interactions are central to uNK-mediated immune regulation at the maternal-fetal interface, with maternal KIR AA genotype combined with fetal HLA-C2 being associated with increased risk of RPL and preeclampsia due to excessive inhibition of uNK cell activity (16). The absence of KIR expression by dNK3 cells suggests that this subset operates outside the classical KIR–HLA-C recognition axis, potentially relying on alternative regulatory mechanisms.dNK2 represents an intermediate phenotype, sharing NKG2A and NKG2C with dNK1 and ITGB2 with dNK3, but lacking the signature markers of either subset (14, 17). This diversification of decidual NK cells reflects the broader functional specialization seen across human NK cell populations (25).

CD103 expression by dNK3 deserves particular attention. CD103 (integrin αE) binds E-cadherin on epithelial cells and marks tissue-resident lymphocytes in mucosal tissues (26). dNK cells expressing CD103 produce more cytokines (GM-CSF, XCL1) and show increased degranulation (CD107a) when stimulated with PMA/ionomycin (17, 27). However, this supraphysiological stimulus may not accurately reflect *in vivo* responses, and the physiological significance of CD103 expression by dNK3 cells in regulating trophoblast interactions requires further investigation. At the transcriptional level, dNK3 cells express several characteristic genes including CCL5, CXCR4, XCL1, CD160, KLRB1, GZMK, *IFNG*, *TNF*SF14, and TIGIT. Together, these genes form a molecular signature that distinguishes dNK3 from other dNK subsets (14, 17).

### 2.2 Transcription factor profile: T-bet-high, eomes-intermediate

The transcription factor landscape of dNK3 cells provides insight into their developmental programming and functional orientation. CyTOF profiling by Huhn et al. demonstrated that dNK3 cells are Eomes-intermediate/T-bet-high, in contrast to dNK1 cells which are Eomes-high/T-bet-low (17). This reciprocal T-bet/Eomes expression pattern parallels the distinction between ILC1 (T-bet-dependent) and conventional NK cells (Eomes-dependent) in other tissue contexts (23, 24), further supporting the classification of dNK3 as an ILC1-like population. Single-cell transcriptomic analysis reveals that dNK3 cells exhibit high expression of TBX21 (encoding T-bet), while dNK1 and dNK2 are enriched for distinct transcription factor programs, including ZNF683 (Hobit) in dNK2 (17, 27).

### 2.3 The dNK3/ieILC1 relationship

A notable feature of dNK3 cells is their close resemblance to intraepithelial type 1 innate lymphoid cells (ieILC1). Huhn et al. provided the most comprehensive evidence for this similarity through multi-parameter CyTOF analysis. They demonstrated that dNK3 cells (clusters c5 and c8 in their analysis) share the CD160^+^NKp44^+^T-bet-high phenotype with ieILC1 characterized in the intestinal epithelium (17). Spatial and temporal mapping of human ILCs across tissues by Yudanin et al. revealed that tissue localization differentially impacts ILC1 distribution and transcriptional profiles, with tissue-specific distinctions particularly apparent for ILC1 populations (28). Cheung et al. demonstrated that iPSC-derived NK cells transcriptionally resemble primary dNK cells. Computational mapping showed that untreated iPSC-NK distributed across dNK subsets with 42% mapping to dNK3, 32% to dNK2, and 22% to dNK1, while TGF-β treatment dramatically shifted the population toward a dNK2-like phenotype (86%) characterized by ZNF683 expression (27). Given the substantial phenotypic overlap, several authors have adopted the dual nomenclature ‘dNK3/ieILC1’ (17, 22, 28), though this usage should be understood as descriptive rather than definitive until lineage tracing or other developmental studies resolve the question.

### 2.4 Comprehensive molecular comparison

**Table 1** compares the molecular and functional characteristics that distinguish dNK1, dNK2, and dNK3 subsets. Data are compiled from multiple studies using scRNA-seq, CyTOF, flow cytometry, and iPSC-based differentiation approaches (14, 17, 20, 27). Important caveat: proportions are approximate and vary across cohorts; differences in gestational age, methodology, and individual variation contribute to this heterogeneity.

**Table 1:** Molecular and functional characteristics of dNK subsets.

Feature	dNK1	dNK2	dNK3
Proportion (term BP)*	~6%	~23%	~71%
Key positive markers	CD39 (high), KIR (high), LILRB1, NKG2A (high), NKG2C	ANXA1, CD18/ITGB2, NKG2A, NKG2C	CD160, CD161/KLRB1, CD103, TIGIT, CD69 (high), NKG2D (high)
Transcription factors	Eomes-HIGH, T-bet-LOW	Eomes (intermediate), T-bet (intermediate)	Eomes (intermediate), T-bet (high)
Top marker genes	*CSF1, ENTPD1, SPINK2*	*ANXA1, ITGB2, ZNF683*	*CCL5, CXCR4, XCL1, IFNG*
Functional profile	Tolerance, angiogenesis, KIR-mediated regulation	Intermediate effector, integrin-mediated adhesion	Polyfunctional cytokine/chemokine production, cytotoxicity
Response to PMA/I	Low (muted)	Intermediate	Highest (CD107a, GM-CSF, XCL1, IFN-γ)
Tissue equivalent	—	—	ieILC1 (gut, liver) [putative]
iPSC-dNK (no TGF-β)	~22%	~32%	~42%
iPSC-dNK (+TGF-β)	~12%	~86%	~1%

Data from Vento-Tormo et al., 2018 (14), Huhn et al., 2020 (17), Jia et al., 2024 (20), and Cheung et al., 2025 (27)(bioRxiv preprint).

*Proportions shown are for term basal plate (BP) from Cheung et al., 2025 (27). First trimester decidua shows different distributions: dNK1 ~30-40%, dNK2 ~45%, dNK3 ~15-20% (Vento-Tormo et al., 2018 (9)). iPSC-dNK proportions represent computational mapping to reference dNK subsets after differentiation with or without TGF-β treatment (Cheung et al., 2025 (27)).

high, strongly positive; intermediate, moderate expression; low, weak expression; negative, absent or minimal expression; BP, basal plate.

## 3 Functional roles of dNK3 cells in normal pregnancy

### 3.1 Ligand-receptor interaction networks at the maternal-fetal interface

**Figure 2:**
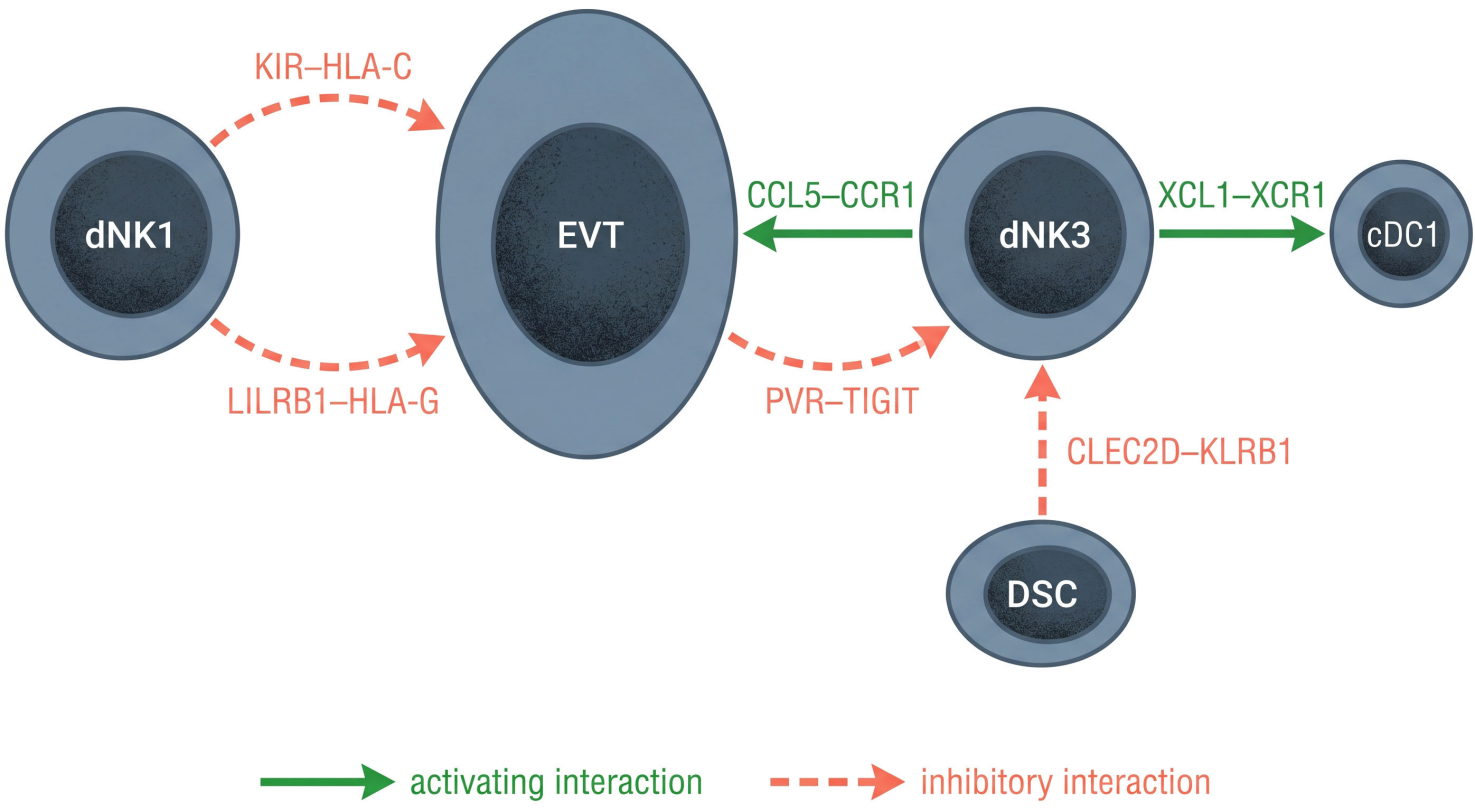
Ligand-receptor interaction networks at the maternal–fetal interface. dNK1 cells engage EVT through KIR–HLA-C and LILRB1–HLA-G inhibitory axes. dNK3 cells participate in chemokine signaling, lectin-mediated regulation, and immune checkpoint modulation (detailed in **Figure 3**). Green solid arrows, activating interactions; red dashed arrows, inhibitory interactions.

dNK3 cell function in the decidual microenvironment has been characterized mainly through CellPhoneDB predictions of ligand-receptor interactions (21), with some protein-level validation (14, 18). CellPhoneDB predictions are based on co-expression of ligand-receptor pairs and represent potential interactions; direct functional evidence for most of these axes in decidua remains limited. Unlike dNK1 cells, which engage extravillous trophoblast (EVT) primarily through inhibitory KIR–HLA-C recognition — a critical axis for pregnancy success (14, 29) — dNK3 cells are predicted to participate in distinct communication axes that emphasize chemokine signaling, lectin-mediated interactions, and immune checkpoint modulation (**Figure 2**). EVTs also express HLA-G, which interacts with ILT2/LILRB1 and KIR2DL4 on dNK cells to suppress cytotoxicity and promote a tolerogenic phenotype conducive to successful implantation (30).

#### 3.1.1 The CCL5–CCR1 axis: potential regulation of EVT migration

The chemokine CCL5 (RANTES) is a characteristic marker gene of dNK3 cells (14, 17). CellPhoneDB analysis of the first-trimester decidua predicted that CCL5 derived from dNK3 cells interacts with CCR1 expressed on EVT, suggesting a paracrine signaling axis that may modulate trophoblast migration and invasion (14, 29). Because trophoblast invasion is essential for spiral artery remodeling and placental perfusion, the predicted CCL5–CCR1 axis warrants functional validation (31, 32), this prediction warrants attention. Direct functional validation of CCL5-mediated EVT migration by dNK3 cells has not been reported. CCL5 is an inflammatory chemokine that can recruit immune effectors and amplify inflammation when present in excess — suggesting dNK3-derived CCL5 may act as a physiological regulator under normal conditions but contribute to pathology when dysregulated (29, 33).

#### 3.1.2 The XCL1–XCR1 axis: crosstalk with dendritic cells

dNK2 and dNK3 subsets produce significantly higher levels of XCL1 (lymphotactin) than dNK1 upon stimulation (17). XCL1 is the sole ligand for XCR1, a chemokine receptor selectively expressed by conventional type 1 dendritic cells (cDC1) in the decidua (14, 17). CyTOF functional profiling shows that dNK3 cells produce the most XCL1 among all dNK subsets after PMA/ionomycin stimulation (17). dNK3 cells can thus bridge innate and adaptive immunity by recruiting and activating cDC1 in the decidual microenvironment (22, 34), though direct functional evidence for this axis in decidual immune regulation is lacking.

#### 3.1.3 The KLRB1–CLEC2D axis: inhibitory regulation

dNK3 cells characteristically express high levels of KLRB1 (CD161), a C-type lectin receptor that binds to CLEC2D (LLT1) expressed by decidual stromal cells and trophoblasts (14, 17). KLRB1 functions as an inhibitory receptor, and its engagement by CLEC2D can suppress inflammatory responses and restrain NK cell effector functions (14, 17). This axis may represent a stromal- and trophoblast-mediated regulatory mechanism to control dNK3 effector responses in the decidual microenvironment.

#### 3.1.4 The TIGIT–PVR axis: checkpoint-mediated inhibition

**Figure 3:**
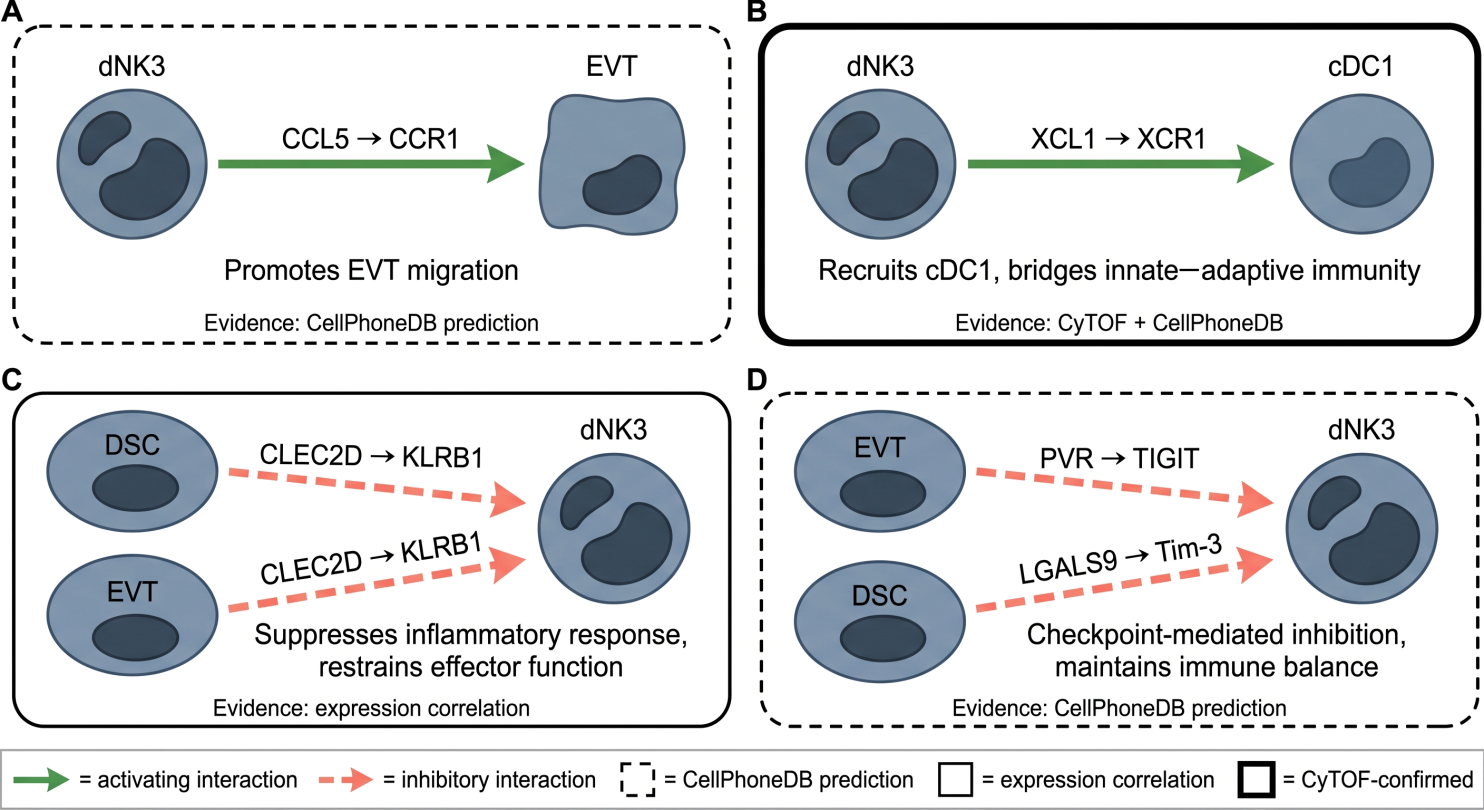
dNK3-specific ligand-receptor interaction axes. Four pathways with functional consequences: CCL5–CCR1 (EVT migration), XCL1–XCR1 (cDC1 recruitment), CLEC2D–KLRB1 (stromal/EVT-mediated inhibition), PVR–TIGIT and Tim-3–LGALS9 (checkpoint restraint). Evidence levels indicated by panel outline style: dashed, CellPhoneDB prediction; solid, expression correlation; bold, CyTOF-confirmed.

TIGIT is an inhibitory immune checkpoint receptor expressed by dNK2 and dNK3 cells (17). Its ligand PVR (CD155) is expressed on extravillous trophoblasts (17), creating a checkpoint axis that may counterbalance the high effector potential of these dNK subsets. Additionally, Tim-3 (HAVCR2) is expressed by subsets of dNK cells and may contribute to immune regulation through interaction with its ligand LGALS9 on decidual stromal cells (14). These inhibitory pathways may help maintain immune balance at the maternal-fetal interface while preserving the capacity to respond to infection, though direct functional evidence in the decidual context remains limited (**Figure 3**).

### 3.2 Signaling pathway characteristics

dNK3 cells exhibit a distinctive signaling pathway architecture. Transcriptomic analysis revealed enrichment of inflammatory signaling pathways, including *TNF* and NF-κB pathways, consistent with dNK3 being the most inflammation-competent subset within the dNK compartment (11). However, it is important to note that pathway enrichment analysis of transcriptomic data reflects transcriptional potential rather than demonstrated functional activity. In contrast, dNK1 cells are enriched for angiopoietin receptor Tie2-mediated signaling pathways that support angiogenesis and vascular remodeling (18). This signaling dichotomy — angiogenic (dNK1) versus inflammatory (dNK3) — represents a putative functional polarization within the dNK compartment (18).

### 3.3 Effector function profile

CyTOF-based functional profiling by Huhn et al. established dNK3 as the most responsive dNK subset under *in vitro* stimulation (17). Following PMA/ionomycin stimulation, dNK3 cells produce significantly higher levels of GM-CSF, XCL1, IFN-γ, MIP-1α (CCL3), and MIP-1β (CCL4) compared to dNK1 cells (17). Many responding dNK3 cells simultaneously produce multiple cytokines, demonstrating polyfunctional effector capacity (17).

The absence of CD39 on dNK3 cells is functionally significant. CD39 is an ectonucleotidase that hydrolyzes extracellular ATP to AMP; together with CD73, this enzymatic cascade generates immunosuppressive adenosine in the decidual microenvironment (20, 35). dNK1 cells express CD39 and exhibit tolerogenic functions, while CD39-negative dNK3 cells lack this immunosuppressive mechanism (20, 35). Jia et al. demonstrated through adoptive transfer in NOG mice that CD56+CD39+ dNK cells (enriched for dNK1) support fetal survival through M-CSF-mediated trophoblast differentiation, while the CD39-negative fraction (enriched for but not exclusively consisting of dNK3) failed to confer such protection (20).CD39 positivity and negativity serve as surrogate markers for dNK1 and dNK3, respectively, but do not constitute pure subset isolation; these fractions likely contain heterogeneous populations.

### 3.4 Gestational and spatial dynamics

Cheung et al. identified spatial heterogeneity in dNK subtype distribution at term. Reference mapping of term scRNA-seq data showed dNK3 cells comprise 71% of NK cells in the basal plate (BP), at term — a marked increase from the dNK1-dominant composition observed in first-trimester deciduas (14, 17), whereas dNK2 predominates in the chorioamniotic membrane (CAM, 57%) (27). Flow cytometry confirmed CD18 (ITGB2) expression was ~5-fold higher in term BP (93.0%) compared to first trimester (17.6%), and CD103 was expressed by ~55% of NK cells in term BP (27). This spatial compartmentalization at term may reflect gestational-stage-specific functional requirements of dNK subsets (27). In preterm labor placentas, dNK3 decreased in BP but increased in CAM, suggesting abnormal spatial redistribution may contribute to preterm labor (27). These data come from a single preprint and need independent validation.

### 3.5 Developmental origins and differentiation trajectories

Guo et al. employed Palantir pseudotime analysis to model dNK differentiation trajectories from dNKp progenitors (18). RNA velocity analysis supported this model, indicating directional differentiation from progenitors to the three mature subsets (dNK1, dNK2, dNK3) (18). TGF-β may function as a negative regulator of dNK3 differentiation: in the iPSC-dNK model, cells without TGF-β yielded 42% dNK3, while TGF-β treatment reduced dNK3 to only 1% and promoted dNK2 to 86% (27). If confirmed, this would suggest that decidual TGF-β actively suppresses dNK3 to maintain the dNK1-dominant composition of healthy first-trimester pregnancy (27). However, it remains uncertain to what extent iPSC-derived dNK cells faithfully recapitulate the differentiation dynamics of primary decidual NK progenitors. Additionally, the TGF-β data specifically await peer-reviewed publication.

## 4 dNK3 in recurrent pregnancy loss: evidence for a subtype imbalance

### 4.1 RPL: immune pathology at the maternal-fetal interface

Recurrent pregnancy loss (RPL), defined as the loss of two or more consecutive pregnancies before 20 weeks of gestation, affects 1–5% of couples worldwide (36). The underlying cause remains unexplained in approximately 40–60% of cases (unexplained RPL, URPL), despite comprehensive evaluation including karyotyping, uterine anatomy, endocrine, and thrombophilia screening (36, 37). The disproportionately high frequency of unexplained cases has long suggested an immunological basis, and the decidual immune microenvironment has emerged as a focus of investigation (18, 33). Early studies documented elevated uterine NK cell density and altered cytotoxicity in women with RPL (38), but the advent of single-cell technologies has revealed that subtype composition, rather than total NK cell numbers, appears to be more consistently altered (18, 39).

### 4.2 The dNK1-Down/dNK3-up shift: cross-study evidence

Guo et al. performed scRNA-seq of 18,646 CD45+ decidual leukocytes from 9 RPL patients and 15 healthy controls (18). Total dNK showed only a slight increase in RPL, but subtype composition changed substantially: dNK1 decreased, dNK2 increased slightly, and dNK3 increased (18). Flow cytometry in an expanded cohort (n = 47) confirmed these changes: dNK1 decreased (P = 0.0004), dNK2 increased (P = 0.0001), and dNK3 increased (P = 0.0019) (18). Chen et al. reported consistent findings using scRNA-seq and flow cytometry, showing KIR2DL1+CD59+ dNK (corresponding to dNK1) reduced from ~40% to ~20% in URPL (39). Wang et al. confirmed lower dNK1 and higher dNK3 in recurrent miscarriage across five NK subclusters (19).

Multiple independent studies have shown consistent results, but several methodological limitations need consideration. First, sample sizes are modest; the largest flow cytometry cohort had 47 subjects (18). Second, as noted in Section 1.2, different groups used different clustering strategies, and ‘dNK3’ in one study may not match ‘dNK3’ in another. Chen et al. defined subsets using KIR2DL1 and CD59, which only partially matches the Vento-Tormo classification (39). Wang et al. (19) identified five NK subclusters rather than three. Third, all studies are cross-sectional, comparing RPL decidual tissue (obtained after diagnosis of pregnancy loss) with decidual tissue from healthy ongoing pregnancies (obtained from elective terminations for non-medical reasons). These controls differ from RPL in many ways beyond immune status (gestational timing, procedure indication, medication exposure, and the physiological state of ongoing vs. failed pregnancy), creating confounding variables. Large prospective cohorts with standardized gating and pre-registered analyses are needed to establish the robustness and clinical utility of these findings.Furthermore, the immunological consequences of the dNK1-down/dNK3-up shift may vary depending on maternal KIR genotype. In KIR AA women, dNK1 depletion would further reduce KIR-mediated inhibitory signaling, potentially compounding the pro-inflammatory effects of dNK3 expansion (16).

### 4.3 Aberrant differentiation trajectory

Beyond static proportion changes, Guo et al. investigated the developmental relationships among dNK subsets using Palantir pseudotime analysis (18). This analysis identified three distinct differentiation paths from dNKp progenitors to mature dNK subsets, with Path 3 being RPL-specific and leading to the dNK3 phenotype (18). scATAC-seq profiling revealed differential chromatin accessibility at key loci such as *IFNG* between dNK subsets (18). Path 3 cells showed enriched expression of cytokine-mediated signaling pathway genes (*IFNG*, *TNF*), while Path 2 cells expressed TGFB1, NFKB1, and REL (18). These findings suggest altered differentiation patterns may contribute to the RPL-associated dNK composition change (18). However, pseudotime analyses are computational inferences based on transcriptomic snapshots and do not constitute direct evidence of cellular lineage relationships. Prospective lineage tracing would be required to demonstrate the proposed differentiation paths.

### 4.4 Molecular abnormalities: *IFNG* dysregulation

RPL dNK3 cells show *IFNG* upregulation at multiple levels. Guo et al. found increased chromatin accessibility at the *IFNG* locus by ATAC-seq, elevated *IFNG* mRNA by scRNA-seq, and increased IFN-γ protein by intracellular staining in RPL dNK3 (18). This pattern — from chromatin to transcription to protein — indicates stable regulation rather than transient responses (18). RPL dNK3 cells also show CD69 upregulation and IRF9 increase (19). Jia et al. found dNK cells from RPL patients had elevated *IFNG* and *TNFA*, reduced HGF and VEGFC, and higher cytotoxicity against JEG3 trophoblast cells compared to normal pregnancy dNK (20). Transplanting RPL patient dNK into NOG mice caused adverse pregnancy outcomes, which improved with co-transfer of CD56+CD39+ dNK and recombinant M-CSF (20). The NOG mouse model has limitations including incomplete immune reconstitution, xenogeneic context, and absence of a fully functional murine immune system, which may limit translational applicability.

### 4.5 Crosstalk with the broader decidual immune network

RPL patients show a broader pattern of immune dysregulation beyond dNK3 expansion. Decidual macrophages were significantly reduced in these patients, particularly the M2-like mac2 subset (18, 40, 41). Using CellPhoneDB analysis, studies have found that macrophages in RPL patients interact with Th1-like T cells through *TNFSF14–TNFRSF14, TNFSF14–LTBR*, and CCL5–CCR1 pairs (18, 21). T cells were elevated and showed Th1 enrichment, matching previous reports of Th1/Th2 imbalance in RPL (18, 42). The dNK subtype shift appears linked to altered immune crosstalk among macrophages, T cells, and stromal cells (18, 33). However, these cross-sectional data cannot determine whether dNK3 expansion drives this immune dysregulation or results from it.The broader alloimmune landscape of RPL extends beyond dNK subtype shifts to encompass Treg/Th17 imbalance, aberrant HLA-G-mediated signaling, and B cell subpopulation changes, underscoring the multi-layered nature of immune dysfunction in this condition (30).

## 5 Diagnostic and Therapeutic Implications

### 5.1 dNK subtype profiling as a candidate diagnostic biomarker

The reproducibility of the dNK1-down/dNK3-up shift across independent cohorts positions the dNK1/dNK3 ratio as a promising diagnostic biomarker for immune-mediated RPL (18, 39, 43). The magnitude of change is substantial—approximately 2-fold—and readily detectable using conventional flow cytometry, with multiple research groups confirming this observation (18, 39). CD39 expression provides clear discrimination between dNK1 (CD39+) and dNK3 (CD39–) populations, enabling a streamlined single-marker approach for clinical flow cytometry panels (20).

Nevertheless, several significant barriers impede clinical translation. The biomarker necessitates decidual tissue acquisition through invasive biopsy. While endometrial biopsy during non-pregnant cycles represents a potential surrogate, the correspondence between non-pregnant endometrial NK subsets and first-trimester decidual NK composition remains uncharacterized. Diagnostic thresholds remain undefined; no study has performed receiver operating characteristic analysis or established sensitivity and specificity for RPL outcome prediction. Intra-individual variability across menstrual cycles has not been quantified. Moreover, a critical confounding factor persists: RPL specimens derive from miscarriage events whereas control specimens originate from elective terminations, introducing gestational age disparities that have yet to receive adequate methodological attention.

### 5.2 Therapeutic strategies: from preclinical evidence to conceptual possibilities

dNK3 research has identified potential therapeutic targets. These strategies range from preclinical proof-of-concept to purely conceptual, with evidence stages noted for each.

#### 5.2.1 M-CSF supplementation (preclinical proof-of-concept)

Jia et al. showed that recombinant human M-CSF (0.5 mg/kg/day, i.p.) significantly rescued adverse pregnancy outcomes in NOG mice adoptively transferred with dNK cells from RPL patients (20). Mechanistically, CD56+CD39+ dNK cells (enriched for dNK1) were identified as an important source of M-CSF at the maternal–fetal interface, whereas dNK cells from RPL patients secreted lower levels of M-CSF. M-CSF signaling through M-CSFR on trophoblast lineages promoted trophoblast differentiation toward both invasive and syncytial pathways: neutralization of M-CSF abrogated the pro-differentiation effects of conditioned medium from normal dNK cells, whereas recombinant M-CSF restored these effects in conditioned medium from RPL dNK cells. *In vivo*, M-CSF treatment reduced abortion rates, increased fetal weight, and increased placental expression of invasion- and syncytialization-related genes. Together, these findings support M-CSF as a functional mediator of CD39+ dNK-associated fetal-supportive activity and a candidate therapeutic target. However, translational interpretation remains cautious, as the NOG model is severely immunodeficient, and the efficacy and safety of M-CSF in immunocompetent systems require further validation.

#### 5.2.2 TGF-β pathway modulation (conceptual)

TGF-β treatment reduced dNK3 in the iPSC-dNK model (27), suggesting pharmacological redirection of progenitor differentiation. This observation comes from a single preprint using an *in vitro* model. TGF-β regulates fibrosis, immunosuppression, and cancer; systemic or local administration carries substantial risk of off-target effects. Therapeutic development requires extensive safety assessment.

#### 5.2.3 iPSC-dNK cell therapy (early preclinical)

The iPSC-dNK platform generates functional dNK cells matching primary dNK transcriptomes (27). Allogeneic NK cell therapies, including iPSC-derived NK cells, have demonstrated safety in cancer clinical trials (44), establishing precedent for allogeneic NK cell therapy. Adaptation for reproductive immunotherapy faces challenges: achieving correct subset composition, ensuring decidual homing, and demonstrating efficacy in physiologically relevant models.

#### 5.2.4 Existing empirical therapies: need for mechanistic reassessment

Existing empirical therapies for RPL—intravenous immunoglobulin (IVIG) (45), corticosteroids, and intralipid infusion—affect both tolerogenic dNK1 and pro-inflammatory dNK3 functions non-selectively (33, 46). Meta-analyses of IVIG for RPL show conflicting results (38, 45), likely because patient selection ignored dNK subtype status. Systematic reviews of immunomodulatory therapies for RPL have reported modest benefits for progesterone (OR 1.38 for live birth), significant improvement with G-CSF (live birth rate 82.8% vs 48.5%), and preliminary efficacy for TNF-α antagonists in combination regimens, yet none of these trials incorporated dNK subtype stratification (47). Future clinical trials need dNK subtype monitoring to assess differential effects on dNK1 versus dNK3 populations and enable better patient stratification (12, 30, 33).Integrating dNK subtype profiling with KIR–HLA-C genotyping may enable more precise identification of patients most likely to benefit from immunomodulation (16).

## 6 Unresolved questions and future directions

### 6.1 Causality versus consequence: a central unresolved question

The most critical unresolved question is whether the dNK1-down/dNK3-up shift causes or results from pregnancy failure. All human studies to date are cross-sectional, comparing tissue from miscarriage with control tissue from elective terminations. This design cannot distinguish three possibilities: (1) the subtype shift causes pregnancy failure by creating a hostile immune environment; (2) the failing pregnancy causes the subtype shift through altered cytokines or tissue damage signals; or (3) both the subtype shift and pregnancy failure stem from a shared upstream cause (e.g., endometrial defect, hormonal imbalance). The NOG mouse data (20) support a causal role of dNK composition, but the xenogeneic and immunodeficient context limits extrapolation. Prospective temporal profiling from pre-conception through early pregnancy to outcome would address causality but is technically challenging. The iPSC-dNK model provides opportunities for *in vitro* testing of whether the RPL microenvironment actively drives pathological differentiation (18, 20, 27).

### 6.2 Nomenclature and identity: the dNK3/ieILC1 question

dNK3 and ieILC1 may be identical cell types or may share a convergent phenotype driven by tissue microenvironments (17, 22–24). Both populations are CD160+T-bet-high and produce IFN-γ upon stimulation, but their developmental relationship is unclear (17, 28). Multi-tissue single-cell analyses with scATAC-seq and lineage tracing can establish whether they are equivalent or distinct (18, 43, 48, 49).

### 6.3 Cross-study comparability and dNK3 heterogeneity

A significant challenge in synthesizing the dNK3 literature is the variability in clustering approaches across studies. Vento-Tormo et al. (14) identified three dNK subsets, Wang et al. (19) identified five, and Huhn et al. (17) found two dNK3 sub-populations (clusters c5 and c8) with distinct marker intensity patterns. The dNK4 subset identified in some studies appears to fall between dNK1 and dNK3 on the differentiation trajectory (19). These discrepancies may reflect genuine biological heterogeneity, differences in computational resolution, or both. Standardization of clustering approaches, benchmarking against well-defined reference datasets, and reporting of full marker panels rather than selected markers would improve cross-study comparability. Higher-resolution profiling using CITE-seq (simultaneous transcriptome and epitope measurement) may help resolve functionally distinct dNK3 sub-states (17, 19).

### 6.4 Spatial organization at the maternal-fetal interface

scRNA-seq destroys spatial context during tissue dissociation. Spatial transcriptomics (Visium, MERFISH, CODEX) (50, 51) applied to decidual sections can reveal where dNK3 cells localize relative to spiral arteries, glandular epithelium, and the trophoblast invasion front (27, 29). This matters because dNK3 activity depends on spatial proximity to target cells.

### 6.5 The missing murine counterpart

No murine uNK subset convincingly corresponds to human dNK3 (17, 22, 33). The NOG xenograft model used by Jia et al. (20) provides proof-of-concept but has fundamental limitations: incomplete immune reconstitution and xenogeneic context. Identifying functionally equivalent murine subsets or developing advanced humanized mouse models is a priority for rigorous mechanistic studies and therapeutic testing (51).

### 6.6 Epigenetic and metabolic regulation

ATAC-seq data revealed altered chromatin accessibility at the *IFNG* locus in RPL dNK3 (18). Comprehensive epigenomic profiling (ChIP-seq, DNA methylation, Hi-C) would reveal the full regulatory logic governing dNK3 identity and pathological activation (49, 52). Metabolic programming—including CD39-related purinergic signaling, glycolytic versus oxidative phosphorylation balance, and lipid metabolism—may represent additional regulatory layers (13). The CD39/CD73 adenosine pathway represents a particularly promising target given its differential expression between dNK1 and dNK3 (20).

### 6.7 Multi-omic integration and computational modeling

Integration of scRNA-seq, scATAC-seq, CyTOF, spatial transcriptomics, and secretomics data through computational frameworks represents an important future direction (53). Single-cell regulatory network inference using methods such as SCENIC (54) has identified candidate dNK3 regulons; expanding this to include chromatin accessibility and protein-level data will provide a more complete picture. These approaches should be complemented by traditional functional validation to ensure that computational predictions translate to biological reality (18, 21, 27).

## 7 Conclusion

Single-cell transcriptomics has established dNK3 cells as a molecularly distinct decidual NK subset with emerging clinical relevance to recurrent pregnancy loss (RPL). The reproducible dNK1-down/dNK3-up shift observed across multiple independent cohorts, coupled with chromatin-to-protein IFNG upregulation in dNK3 cells, positions this subset imbalance as a candidate diagnostic biomarker. Preclinical investigations have identified M-CSF supplementation and TGF-β pathway modulation as potential therapeutic targets, though rigorous validation in immunocompetent systems remains requisite. Critical questions persist regarding causality versus consequence, cross-study comparability, and the developmental relationship between dNK3 and ieILC1. While the current evidence base is insufficient to warrant clinical translation, dNK subtype profiling may serve as a stratification tool in future interventional trials. As the field advances, dNK3 cells hold promise as central mediators in precision medicine approaches to pregnancy complications.
